# Retraction: MiR-153 regulates expression of hypoxia-inducible factor-1α in refractory epilepsy

**DOI:** 10.18632/oncotarget.28811

**Published:** 2025-12-31

**Authors:** Guo-Hua Gong, Feng-Mao An, Yu Wang, Ming Bian, Di Wang, Cheng-Xi Wei

**Affiliations:** ^1^Medicinal Chemistry and Pharmacology Institute, Inner Mongolia University for the Nationalities, Tongliao, Inner Mongolia, P.R. China; ^2^Inner Mongolia Key Laboratory of Mongolian Medicine Pharmacology for Cardio-Cerebral Vascular System, Tongliao, Inner Mongolia, P.R. China; ^3^First Clinical Medical of Inner Mongolia University for Nationalities, Tongliao, Inner Mongolia, P.R. China; ^*^These authors contributed equally to this work

**This article has been retracted:** Oncotarget has completed the investigation of this paper following the retraction request of the corresponding author Chengxi Wei. During our review, we became aware that three authors—Chengxi Wei, Guohua Gong, and Fengmao An—were investigated by the National Natural Science Foundation of China (NNSFC). NNSFC concluded that Chengxi Wei “had problems with buying and selling experimental data, fabricating research processes, and improper authorship,” a translation of the report states. Wei had two projects revoked and was permanently banned from NSFC projects. Guohua Gong and Fengmao An were “responsible for the problems of buying and selling experimental data and fabricating the objective results of the research process in the relevant papers, as well as the abuse of project funds.” Additionally, we received a letter from Yu Wang, an author listed on the paper, who stated that he/she was not involved in the publication. The Office of Scientific Integrity attempted to contact all listed authors multiple times to gather detailed information and obtain agreement for the retraction. These efforts were unsuccessful, as we received no replies or the emails were returned as undeliverable. Based on the findings of these investigations, the Editorial decision has been made to retract the article.

Original article: Oncotarget. 2018; 9:8542–8547. 8542-8547. https://doi.org/10.18632/oncotarget.24012

